# Hands‐on radiotherapy: Inspiring the next generation of STEM students

**DOI:** 10.1002/acm2.12595

**Published:** 2019-05-02

**Authors:** James Patrick, Alice Colthurst, Madura Nanthakumaran

**Affiliations:** ^1^ Medical Student Imperial College London London UK

Dear Editor,

We read with interest the article by Fagerstrom et al.^1^ which explored an outreach project aimed at increasing awareness of the role of medical physicists amongst middle school students.

Despite being medical students in our penultimate year of study in the UK, we have incredibly limited knowledge of careers involving medical physics and radiation therapy. Only within the last few months have we had our first interactions with these healthcare professionals. We believe that this is an area of medicine which is poorly covered, if not absent, in the university curriculum, let alone at middle school level. On reflection, we feel it is of limited utility to discover the existence of these career opportunities at such a late stage in our education. If we had known about such career opportunities at a younger age, we may have considered them when applying to university; therefore, the outreach project produced by Fagerstrom et al. should be commended, and we would like to see this approach replicated both nationally and internationally.

The concept of bringing undergraduate level education into the school classroom has already been explored in the UK. *Authentic Biology* is a program which has established student‐led undergraduate‐level research projects in seven schools across the UK, with the aim of inspiring the next generation of biomedical researchers.^2^, ^3^ With funding from the Wellcome Trust, and through collaborations with local universities, this project has inspired students to consider medical research by allowing them to experience these careers before enrolling on a university course. Though aimed at slightly older students (16–18‐year‐old) this project doubled the percentage of students interested in biological careers, and 65% of students involved in the initial project have continued to study STEM based courses. We feel that if the outreach project described by Fagerstrom et al. was further developed, it could have a similar impact on developing interest in careers in medical physics, by allowing students to “trial the job” as opposed to learning about it from a career's website.

This approach has wider implications for the way in which we deliver careers advice to school students. A “hands‐on experience” of this kind is likely to be far more relevant and useful to students than an afternoon spent in the school counselor's office.

We believe that more of these outreach projects should be instigated, to inspire the next generation of STEM students.

## CONFLICTS OF INTEREST

For completeness Alice Colthurst would like to acknowledge that she was a student participant in the Authentic Biology project, which was initially developed by her father, Dr David Colthurst.
